# Acoustic Analysis of Vocalizations in Malinois Dogs: Context-Associated Variation in Fundamental Frequency, Harmonic-to-Noise Ratio, and Formants

**DOI:** 10.3390/vetsci13060519

**Published:** 2026-05-27

**Authors:** Baoan Li, Liuwei Xie, Mingqiang Song, He Zhai, Ning Sun, Xiuxiang Meng

**Affiliations:** 1 Department of Police Dog Technology, Criminal Investigation Police University of China, Shenyang 110048, China; libaoan@cipuc.edu.cn (B.L.); xielw195@163.com (L.X.); song15263936064@163.com (M.S.);; 2 School of Ecology & Environment, Renmin University of China, Beijing 100872, China

**Keywords:** dog vocalizations, affective states, acoustic analysis, fundamental frequency, harmonic-to-noise ratio, formants

## Abstract

This study investigated context-associated variation in vocalizations in Malinois dogs through acoustic parameter analysis. Vocalizations from thirty adult Malinois dogs (15 males, 15 females) aged 2 to 3 years were recorded across 11 behaviourally defined contexts. Using Praat software, key acoustic parameters—fundamental frequency (F0), harmonic-to-noise ratio (HNR), and formant frequencies—were extracted and analyzed. Results indicated that different vocalization types (barking, whimpering, growling, snarling, howling) exhibited distinct acoustic profiles. Whimpering and howling showed significantly higher F0 values than barking (*p* < 0.05), with whimpering uniquely displaying both low and high F0 components. Dogs in contexts expected to be positively valenced (e.g., food anticipation) showed lower HNR than those in contexts expected to be negatively valenced (e.g., separation) (*p* < 0.05). However, the actual internal states were not independently verified. Formant analysis revealed that snarling and howling had lower Formant 1 (F1) values (*p* < 0.05), while formant dispersion varied with emotional state. These findings suggest that acoustic analysis of dog vocalizations can provide objective insights into dogs’ motivational and arousal changes, thereby improving our understanding of canine vocal communication, social behavior, and the human–dog bond. This approach has potential applications for working-line Malinois breeding programs and for enhancing human–working dog interactions.

## Simple Summary

Dog vocalizations may reflect inner motivational states, but objective assessment remains challenging. We recorded 30 Malinois dogs across 11 standardized scenarios (e.g., play anticipation, brief separation, stranger approach) designed to elicit varying arousal and putative valence. Using Praat software, we extracted fundamental frequency (F0), harmonic-to-noise ratio (HNR), and formant frequencies. Whines and howls showed higher F0 than barks; vocalizations recorded in positive contexts had lower HNR; and vocalizations from aggressive- or distress-associated contexts exhibited distinct formant patterns. These results indicate that acoustic features co-vary systematically with situational context, providing a non-invasive window into arousal and motivational changes in Malinois dogs. However, independent validation with physiological and behavioral measures is required before these acoustic parameters can be considered reliable indicators of specific behavioral contexts or underlying affective states. This approach holds promise for veterinarians and animal behavior professionals working with working-line dogs.

## 1. Introduction

Recognizing animal emotions is crucial for understanding their responses to internal and external stimuli [1]. Accurately assessing emotions, particularly positive ones, is essential for improving animal welfare [2,3]. Recent years have seen growing interdisciplinary interest in animal emotion research, spanning evolutionary zoology, affective neuroscience, comparative psychology, animal welfare science, and psychopharmacology [1]. However, measuring emotions in non-human animals remains challenging due to the impossibility of accessing their subjective experiences directly. Therefore, indirect indicators such as neurophysiological, behavioral, or cognitive markers are necessary to infer emotional states.

Vocalizations are linked to the inner emotional state of the caller and can serve as non-invasive indicators for assessing animal emotions [3,4]. Unlike humans, animals have limited voluntary control over their vocal apparatus. Emotions induce changes in the somatic and autonomic nervous systems, leading to muscular adjustments in the vocal tract that manifest as specific acoustic alterations [5]. Two acoustic parameters are particularly informative in this context. Formant frequencies are the resonant frequencies of the vocal tract, determined by its length and shape; they convey information about the caller’s body size. The harmonic-to-noise ratio (HNR) quantifies the relative amount of periodic (harmonic) versus aperiodic (noise) energy in a vocalization. Lower HNR values reflect increased irregularity in vocal fold vibration, often associated with heightened emotional arousal or positive affective states in mammals. Morton’s Motivation-Structural (MS) rules predict that acoustic structure varies with context: low-frequency sounds are associated with hostile settings, whereas high-pitched vocalizations occur in fearful or appeasing contexts [6]. These rules have been validated across numerous mammalian species and applied to study emotion-related vocalizations, including in humans [4,7,8]. Emotional shifts often correspond to changes in vocalization type; for example, humans shift from laughter to crying, horses from chirping to screaming, and rats from 22 kHz to 50 kHz ultrasonic calls [9]. Thus, animal vocalizations provide a reliable, non-invasive means of emotion assessment.

Vocalizations are integral to animal cognition, facilitating individual recognition, social interaction, mate selection, predator avoidance, and territorial marking [10,11]. As highly social canids, dogs possess a diverse and complex vocal repertoire that expanded during domestication as they adapted to human social environments [10]. Previous studies have categorized dog vocalizations into types such as barking, growling, whimpers, howling, and their combinations [12,13]. Dogs vocalize differently across emotional states, allowing inferences about their affective state from vocal cues. Traditional methods that depend on subjective auditory assessment are prone to observer bias and lack objectivity. Recent research has thus focused on the bioacoustic characteristics of dog vocalizations, examining parameters such as frequency, duration, vocal type, fundamental frequency (F0), intensity, and spectral energy [14,15,16]. Taylor et al. demonstrated that emotions influence the neural control of laryngeal and vocal tract muscles, leading to changes in acoustic parameters including intensity, duration, F0, and formant frequencies [17].

Bioacoustics—the interdisciplinary study of animal sounds—has advanced rapidly with improvements in recording technology and data analysis [18]. Digital recording, storage, and sharing now facilitate large-scale bioacoustic monitoring and continuous audio capture [19]. Manual analysis, involving auditory inspection and spectrogram examination, still constitutes about 80% of acoustic research methods [20]. Praat, a widely used speech analysis software in linguistics, offers free tools for analyzing, annotating, processing, and synthesizing digitized speech signals [21]. In this study, we used Praat to collect and analyze dog vocalizations, extracting three key acoustic parameters: fundamental frequency, formant frequencies, and HNR. Based on the MS rules and prior research, we hypothesized that: (1) vocalizations recorded in distress-related contexts (whimpering, howling) would show higher F0 than those in excitement-related contexts (barking); (2) vocalizations from contexts with positive valence would show lower HNR than those from contexts with negative valence; (3) aggressive-context (snarling, growling) and distress-context (howling) vocalizations would exhibit distinct formant characteristics. Our objective was to test these hypotheses and explore the relationship between vocalizations and emotional states, thereby establishing an acoustic basis for emotion assessment in dogs.

## 2. Materials and Methods

### 2.1. Subjects and Setup

Thirty adult Malinois dogs (15 males, 15 females) aged 2 to 3 years, all working police dogs from the Police Dog Training Base of the Criminal Investigation Police University of China, participated in the study. Dogs were housed individually in standardized kennels with independent activity spaces and had received consistent basic training (e.g., obedience, bite work) prior to experiments. Each dog was recorded in all 11 scenarios, yielding an average of 76.9 ± 5.3 valid vocalization samples per dog (range: 70 to 85). No significant difference in valid recording number was found between sexes (t = 1.236, Df = 28, *p* > 0.05). The primary recording device was a professional solid-state recorder (TASCAM DR-100MKIII, TEAC Corporation, Japan) equipped with a built-in unidirectional condenser microphone (frequency response: 20 Hz to 20 kHz). Recordings were captured at a 44.1 kHz sampling rate and 16-bit depth. An iPhone 13 Plus was used as a synchronized backup device (backup, 44.1 kHz sampling rate, 16-bit depth). Most recordings were made in a sound-attenuated laboratory (50 m^2^, background noise < 30 dB). Additional locations included: (1) Individual kennels (scenarios C, E, J); (2) A novel office adjacent to the lab (scenario D); (3) An outdoor enclosed interaction area (20 m × 15 m, scenario F). All non-lab sites were pre-screened for background noise (<40 dB). All outdoor recordings were conducted in calm conditions (wind speed < 2 m/s). The microphone-to-dog distance was maintained between 1.0 and 1.5 m; this range does not affect the relative acoustic measures extracted in this study.

### 2.2. Sound Recording in Different Behavioural Contexts

Vocalizations were recorded across 11 standardized scenarios, each designed to evoke different behavioural reactions. Scenarios were conducted on separate days (one per day) in randomized order to avoid carryover effects. Each session lasted 5 min (2 min acclimation + 3 min recording). A. Bite Training: Experimenter waved a bite sleeve without contact; B. Ball Prevention: Tennis ball placed 0.5 m away, inaccessible; C. Feeding: Experimenter approached kennel with food bowl; D. Exploratory Behavior: Dog placed in novel office with unfamiliar objects; E. Alone: Dog left alone in kennel; F. Encounter with Large Dog: A pit bull introduced behind a barrier; G. Stranger Approach: Unfamiliar persons approached kennel; H. Food Removal: Experimenter attempted to remove bone with forceps; I. Ball Retrieval Help: Ball placed in inaccessible hole; J. Owner Away: Owner leashed dog and left room; K. Howling Induction: Pre-recorded howling played back.

### 2.3. Acoustic Collection and Analysis

Recordings (16-bit, 44.1 kHz, WAV format) were analyzed in Praat 6.0. Spectrograms were generated with settings: 0.03 s window length, 0.01 s time step, 250 Hz frequency step, 60 dB dynamic range, 20 Hz frequency resolution, Gaussian window. Fundamental frequency (F0), formants (F1 to F4), and harmonic-to-noise ratio (HNR) were measured from vocal samples across all contexts. Praat’s automated algorithms were used for initial extraction of F0, formants, and HNR. Twenty percent of samples were manually verified by two independent raters; inter-rater agreement for parameter extraction exceeded 95%.

### 2.4. Statistical Analysis

Data were analyzed in GraphPad Prism 5 and expressed as mean ± SD. For each acoustic parameter (F0, HNR, F1–F4, formant dispersion), differences among the 11 scenarios were first examined using one-way ANOVA. Based on the three a priori hypotheses, we defined 14 planned, hypothesis-driven comparisons across the three parameter families: Fundamental frequency (F0, 6 comparisons): whimpering (I) vs. barking (C, E); howling (K) vs. barking (C, E); play-related barking (B, C) vs. threat-related barking (F); Harmonic-to-noise ratio (HNR, 6 comparisons): * positive-context vocalizations (A, B, C) vs. negative-context vocalizations (D, E, F); barking (C) vs. whimpering (I). Formant frequencies (2 comparisons): snarling (H) vs. howling (K) on F1; growling (G) vs. snarling (H) on F4. Independent-samples t-tests were used for these planned comparisons. To control the family-wise error rate, a Bonferroni correction was applied across the 14 tests. For clarity, results are reported as significant at *p* < 0.05 (*) and *p* < 0.01 (**) after this correction. All *p*-values in this study should therefore be regarded as exploratory indicators accompanying the descriptive statistics. Future confirmatory studies should employ linear mixed-effects models with Dog ID as a random factor to appropriately partition within- and between-subject variance. The formula was applied to calculate formant dispersion (Df):

$$Df=\frac{\sum_{i=1}^{N-1}F_{i+1}-F_{i}}{N-1},$$

where Df is formant dispersion, N = 4 (the total number of formants measured, F1 to F4), and Fi is the frequency of the i-th formant. Df thus represents the average spacing between consecutive formants [22].

## 3. Results

**Figure 1:**
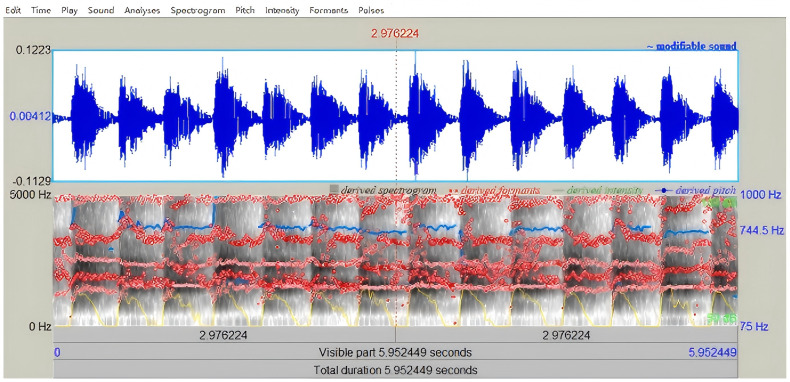
**Representative **spectrogram of a dog whimpering vocalization. The upper panel displays the waveform (time domain). The lower panel displays the spectrogram. In the lower panel, the left y-axis (0–5000 Hz) indicates the frequency scale for the spectrogram (black) and formant tracks (red dots). The right y-axis (75–1000 Hz) indicates the frequency scale for the pitch contour (F0, blue line). The yellow contour represents intensity, with its scale in this figure. Spectrogram settings were: window length 0.03 s, time step 0.01 s, frequency step 250 Hz, dynamic range 60 dB.

Acoustic parameters were extracted and analyzed for all 11 scenarios. Vocalizations were visualized via spectrograms (Figure 1), displaying waveforms (top) and spectrograms (bottom). Dogs produced distinct vocalization types across scenarios (Table 1): barking (A to F), whimpering (I, J), growling (G), snarling (H), and howling (K). Corresponding behaviors included active approach, tail wagging, alert posture, teeth display, and head-up posture.

### 3.1. Fundamental Frequency

**Figure 2:**
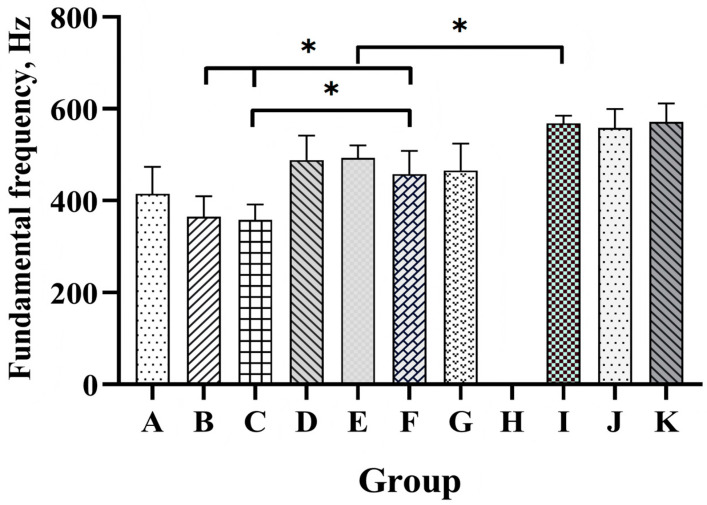
**Fundamental frequency (F0) across the 11 behavioural contexts.** Data are presented as mean ± SD. Scenario H (snarling) yielded no measurable F0 and is omitted. Significance is reported at the *p* < 0.05 level after Bonferroni correction for 14 planned comparisons. Significance is indicated by * (*p* < 0.05 after Bonferroni correction for 14 planned comparisons). Error bars represent Standard Deviation (SD).

Vocalization types were classified independently by two experienced canine behavior analysts. As shown in Table 1 and Figure 2, whimpering and howling exhibited significantly higher F0 than barking. Specifically, Group I (whimpering) had a higher F0 than Group E (barking) (*p* < 0.05, t = 3.292, df = 75) and Group C (*p* < 0.05, t = 5.739, df = 96). Snarling (Group H) consisted predominantly of aperiodic, turbulent noise, precluding reliable F0 detection. Within barking types, play-related barks (Groups B, C) had lower F0 than threat-related barks (Group F) (*p* < 0.05). F0 did not differ significantly between barks within the same emotional context (A vs. B, E vs. F; *p* > 0.05).

**Figure 3:**
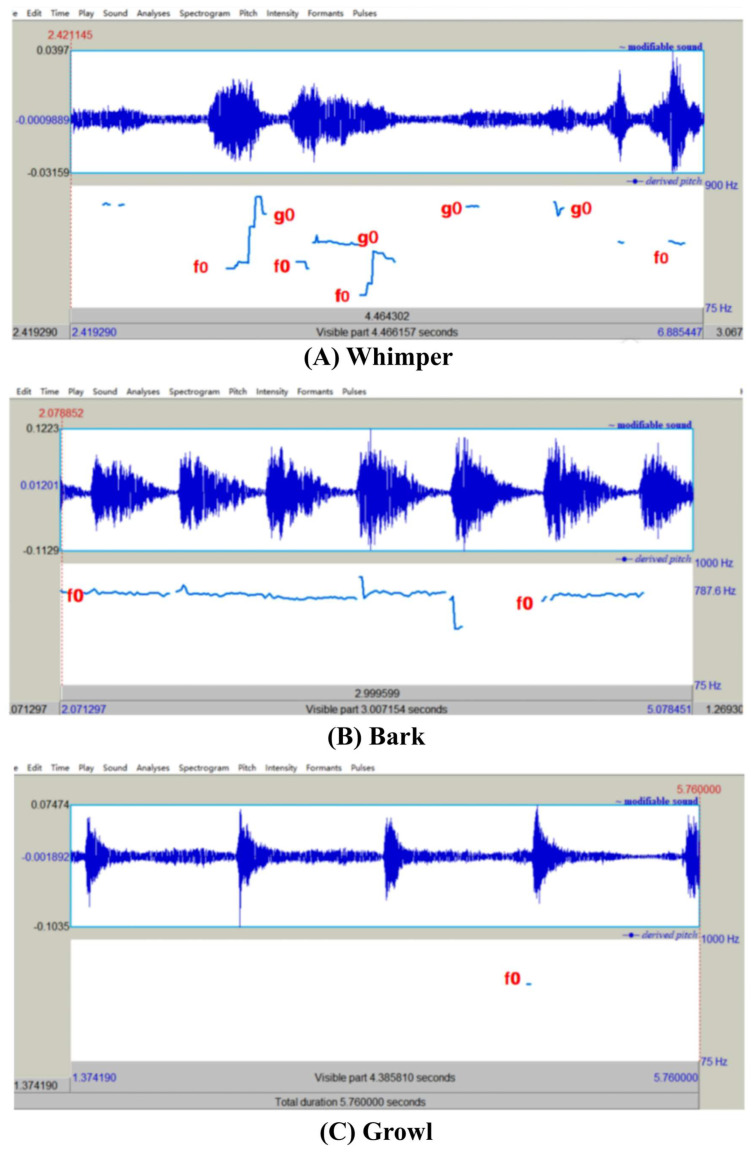
**Fundamental frequency spectrum of three vocalization types**.

Figure 3 illustrates spectrograms for three vocalization types. Barking and growling exhibited regular, short-duration waveforms (0.398 s and 0.169 s, respectively). Whimpering showed an irregular, longer waveform (1.274 s) with amplitude fluctuations and a harsh quality. The F0 of barking remained stable near 787.6 Hz (Figure 3B), whereas whimpering displayed irregular F0 with both low (F0) and high (G0) components, occurring singly, simultaneously, or with frequency jumps (Figure 3A). Growling contained few detectable F0 components (Figure 3C).

### 3.2. Harmonic-to-Noise Ratio

**Figure 4:**
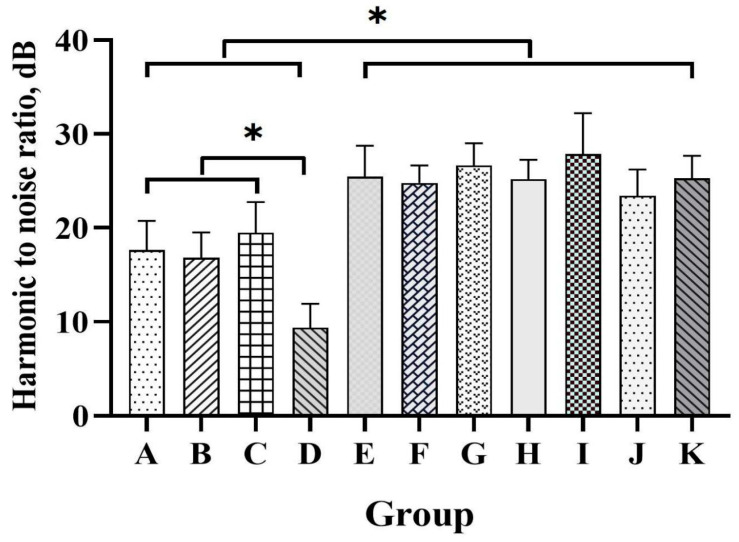
**Harmonic-to-noise ratio (HNR) across the 11 behavioural contexts.** Data are presented as mean ± SD. Letters A–K above the bars correspond to the 11 scenarios defined in Section 2.2: A, bite training; B, ball prevention; C, feeding; D, exploratory behavior; E, alone; F, encounter with large dog; G, stranger approach; H, food removal; I, ball retrieval help; J, owner away; K, howling induction. Significance is indicated by * (*p* < 0.05 after Bonferroni correction for 14 planned comparisons). Error bars represent Standard Deviation (SD).

HNR values varied significantly across groups (Figure 4). Barking groups A to C (excitement) showed higher HNR than Group D (curiosity) (*p* < 0.05), while Group D had lower HNR than Groups E and F (fear) (*p* < 0.05). Among non-bark vocalizations, Group C (barking) had lower HNR than Group I (whimpering) (*p* < 0.05). No significant pairwise differences were found for growling (G), snarling (H), whimpering (J), or howling (K) compared to other groups (*p* > 0.05).

### 3.3. Formants

**Figure 5:**
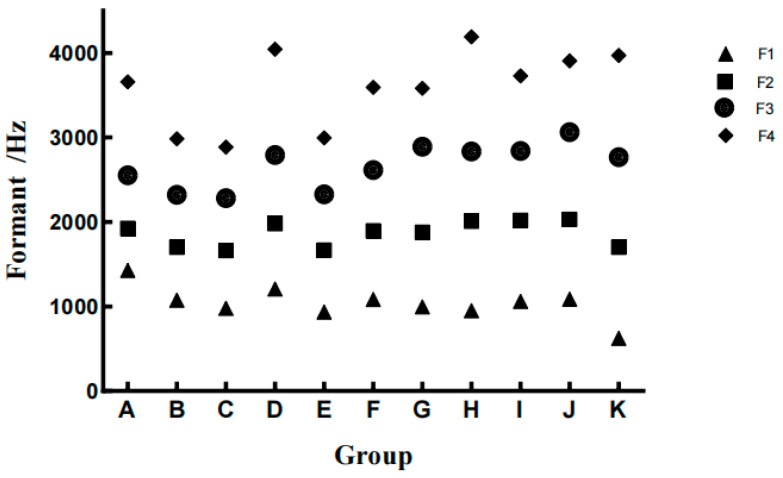
**Formant frequencies across groups.** Abbreviations: F1–F4, first to fourth formant frequencies (0–5500 Hz). Letters A–K on the x-axis refer to the 11 behavioural scenarios detailed in Section 2.2: A, bite training; B, ball prevention; C, feeding; D, exploratory behavior; E, alone; F, encounter with large dog; G, stranger approach; H, food removal; I, ball retrieval help; J, owner away; K, howling induction.

Formant analysis used linear predictive coding (LPC) with a 0–5500 Hz search range and a maximum of four formants tracked per frame (Figure 5). Formant 1 (F1) was lowest in Group K (howling, 623.22 ± 234.73 Hz), followed by Group H (snarling, 950.6 ± 191.21 Hz). Group H also exhibited the highest F4 (4191.21 ± 570.81 Hz). No significant F1 difference existed between growling (G) and snarling (H) (*p* > 0.05), but F4 was significantly lower in Group G (*p* < 0.05). Formant dispersion was denser in Groups A to C (Df = 635.41–743.78 Hz) and more dispersed in Groups H and K (Df = 1080.20 Hz and 1115.77 Hz, respectively).

## 4. Discussion

Researchers have recently investigated various acoustic characteristics of dog vocalizations, including frequency, duration, vocalization type, and parameters such as fundamental frequency (F0), intensity, and spectral energy [14,15,16]. Studies have demonstrated that acoustic features can provide insights into the context and background of dog vocalizations [23], convey information about body size and emotion to humans [24], and enable humans to categorize barking based on contextual variations [25]. However, distinguishing between different emotional states based solely on acoustic parameters remains challenging, primarily due to two factors: (1) Traditional methods rely on subjective auditory judgment, which is prone to inter-observer bias [26]; (2) Overlaps in acoustic features between adjacent emotional states (e.g., excitement vs. curiosity) are common. The current study fills this gap by adopting objective bioacoustic analysis: we extracted quantifiable parameters (F0, HNR, formants) and verified their correlation with standardized emotional scenarios, providing a data-driven basis for distinguishing canine emotional states.

The fundamental frequency (F0) of dog vocalizations is influenced by physiological factors (e.g., gender, age, health status) and psychological factors (e.g., emotion, state of mind). Reported F0 values for dogs vary widely across studies, ranging from 90 to 1010 Hz [27,28,29,30], which is consistent with the F0 range (75.38–599.16 Hz) observed in our study. In interactions with humans, barks and whimpers are common vocalizations, with whimpers typically produced in situations of frustration, loneliness, or attention-seeking. Our experiment revealed that whimpering has a higher F0 than barking, attributed to the presence of a high fundamental frequency (G0) alongside F0. This simultaneous production of low (F0) and high (G0) fundamental frequencies is a distinctive feature of canine sound [31], also observed in other canids, although the mechanism of G0 production remains unclear [32]. It has been suggested that high-frequency whimpers are produced through the nose with the mouth closed, while low-frequency barks are produced through the mouth. The coexistence of high and low frequencies, as well as frequency jumps, was observed in dog whimpers. The high G0 is primarily found in dogs experiencing distress, possibly indicating extreme stress on the vocal apparatus [33]. The inability to extract F0 from snarls is consistent with the highly noisy, chaotic acoustic structure of aggressive vocalizations, which lack periodic vocal fold vibration.

Barking exhibits considerable variability in acoustic characteristics, with a frequency range of 160 to 2630 Hz [11], and differs between breeds and individuals [13]. Barking is short, explosive, and repetitive, contrasting with whimpers that express frustration and pain. Barking occurs in various contexts, such as guarding, competition, defense, interaction, exploration, greeting, or play [34]. Barking also varies with the external environment, particularly the social environment [14]. Human listeners can discriminate barks according to perceived emotion, rating barks directed at strangers as more aggressive, barks emitted during isolation as more distressed, and barks produced during play as happier [25]. Distinguishing between various barks aurally is challenging, but can be achieved through vocal behavior performance and acoustic characteristics [5]. In our experiment, the acoustic characteristics of barking under threat and during play differed, with play barking having a lower F0 than aggressive barking, consistent with Morton’s motivation-structure (MS) rule and previous research showing that positive emotions are associated with lower and less variable F0 [6,7].

The harmonic-to-noise ratio (HNR) compares the amplitude of sound harmonics to the amplitude of noise and varies across situations [35]. HNR and F0 are influenced by mood and can serve as indicators of emotional state [17]. In our experiment, vocalizations recorded in positively valenced contexts (e.g., food anticipation) showed lower HNR than those recorded in negatively valenced contexts (e.g., isolation, threat). Whether this reflects valence or primarily arousal remains to be clarified by future studies incorporating physiological validation, broadly consistent with work showing that humans can detect negative arousal in some non-human vocalizations [36]. Notably, Scenario D (curiosity/fear) exhibited lower HNR than the positive-emotion scenarios A–C, which appears inconsistent with a simple positive-vs-negative valence interpretation. One possible explanation is that HNR is more directly influenced by emotional arousal rather than valence per se; the high arousal of exploration may increase vocal perturbation and reduce HNR. Future studies with independent arousal and valence manipulations are needed to clarify this relationship. Thus, HNR may be a useful acoustic parameter for recognizing positive emotions in dogs, though its sensitivity to arousal warrants caution in interpretation.

In human speech, formants are crucial for phoneme differentiation, particularly formant 1 (F1) and formant 2 (F2) in vowel recognition [37]. However, their role in animal communication is less understood. Some studies suggest that formants may have functional significance in non-verbal animal communication and in the perception of emotions, with lower formants indicating anger [38,39,40]. In our experiment, snarling and howling had lower F1 values. Dogs use snarling to intimidate opponents or intensify attacks, and snarling during food protection is considered highly aggressive. Lower formants may be related to vocal tract length, which can be altered to exaggerate body size in some mammals [41]. Individuals with lower larynxes produce lower formants, providing an advantage in competition and mate selection [42]. Formant dispersion, which reflects vocal tract length and body size [43], may be a reliable cue for dogs to assess the body size of conspecifics. Our experiment revealed changes in formant dispersion with varying emotions, suggesting a potential relationship between formant dispersion and dog emotions that warrants further investigation. The correlation between dog vocalizations and the origin of the vocal tract also requires further study. It should be noted that canid howling is functionally diverse and its acoustic structure may vary with context. In wolves (Canis lupus), howling serves multiple social functions: group howls can function as pre-activity bonding signals (“pep rallies”) before coordinated hunts, as territorial displays to neighboring packs, or as responses to external triggers such as sirens, while solitary howling typically occurs when an individual becomes separated from the group and likely reflects separation distress [12,13]. In our study, howling was elicited by playback of pre-recorded howls (Scenario K). Under this experimental paradigm, the vocalizations obtained may represent a mixture of social cohesion response and territorial reactivity rather than exclusively distress. This functional diversity of howling across canid species warrants further investigation comparing acoustics across different howling-eliciting contexts.

In addition, this study has several limitations: (1) The subjects were exclusively adult working Malinois police dogs. Generalization to other breeds, pet dogs, or different age groups requires independent validation; (2) Only three core acoustic parameters were analyzed, and future studies could include duration, spectral energy, and other parameters. Although scenario order was randomized, potential habituation or sequential effects were not statistically tested; (3) Emotional states were inferred from scenarios rather than verified through physiological indicators (e.g., cortisol levels, heart rate) or systematic behavioral coding. This reliance on context-based emotional attribution introduces a potential circularity risk, as the acoustic differences observed may reflect context rather than emotion per se. Future studies should integrate physiological and behavioral measures to independently validate emotional states. Furthermore, canine communication is inherently multimodal: facial expressions, body postures (e.g., play bows, tail carriage), and vocalizations jointly signal emotional and motivational states [10,34]. For example, growls emitted during playful tug-of-war may be acoustically similar to those produced during genuine resource disputes, with the contextual meaning disambiguated primarily by visual play signals. The present study focused exclusively on the acoustic modality; simultaneous analysis of visual and vocal signals is needed to fully characterize the emotional content of dog communication. Future work should expand the sample size and breed diversity, and develop a machine learning model based on acoustic parameters for automatic emotion recognition in dogs. Additionally, many positive social vocalizations in adult dogs (e.g., greeting whimpers, play-soliciting barks) are considered “socio-infantile” signals derived from puppy vocalizations directed toward the mother or littermates [10,12]. Comparative acoustic analysis of puppy and adult vocalizations would help identify which acoustic features are retained ontogenetically and whether these features serve similar affiliative functions across developmental stages.

## 5. Conclusions

This study provided evidence supporting three hypotheses through acoustic analysis of 30 Malinois dogs across 11 emotional scenarios. Vocalizations recorded during whimpering and howling contexts showed higher F0 than those during barking contexts; calls from positively valenced contexts displayed lower HNR; and snarling/howling exhibited lower F1. These results suggest that F0, HNR, and formants are promising acoustic indicators that differ systematically across emotional contexts in Malinois dogs. However, formal cross-scene classification validation (e.g., via machine learning) is required to establish their reliability as independent diagnostic markers of canine emotional states.

## Figures and Tables

**Table 1:** **Acoustic parameters of dog vocalizations across expected affective context scenarios**.

Scenario	Contexts	Vocalization Type	Fundamental Frequency, Hz			Harmonic to Noise Ratio, dB			Formant, Hz				
			Min	Max	M ± SD	Min	Max	M ± SD	Formant 1	Formant 2	Formant 3	Formant 4	Formant Dispersion
									M ± SD	M ± SD	M ± SD	M ± SD	
A	Excitement	Barking	128.08	535.51	415.67 ± 95.07 ^bc^	5.99	27.09	17.54 ± 2.92 ^b^	1427.1 ± 180.01 ^a^	1923.2 ± 281.46	2552.19 ± 349.83	3658.44 ± 580.65 ^ab^	743.78
B	Excitement	Barking	196.81	595.98	399.88 ± 72.41 ^c^	9.95	18.47	16.35 ± 2.74 ^b^	1075.46 ± 160.41 ^ab^	1702.31 ± 165.65	2321.85 ± 150.44	2984.55 ± 134.4 ^b^	636.36
C	Excitement	Barking	91.78	507.64	359.12 ± 57.9 ^c^	6.65	9.8	19.23 ± 3.23 ^b^	980.4 ± 215.23 ^b^	1661.26 ± 226.51	2282.05 ± 221.49	2886.63 ± 243.63 ^b^	635.41
D	Exploratory, Fear	Barking	332.15	599.21	482.2 ± 100.92 ^ab^	14.71	24.45	9.29 ± 2.92 ^c^	1206.92 ± 263.65 ^ab^	1979.91 ± 289.84	2792.81 ± 342.7	4043.97 ± 464.99 ^a^	945.68
E	Loneliness	Barking	470.92	589.8	496.84 ± 55.51 ^b^	22.69	23.39	24.04 ± 3.49 ^a^	934.25 ± 258.37 ^b^	1667.17 ± 179.62	2327.51 ± 171.92	2995.15 ± 128.9 ^b^	686.97
F	Alertness, Fear	Barking	236.83	596.64	460.01 ± 111.7 ^ab^	20.44	26.71	23.44 ± 1.77 ^a^	1083.31 ± 219.52 ^ab^	1895.51 ± 227.63	2616.52 ± 271.83	3592.65 ± 458.41 ^ab^	836.45
G	Alertness	Growling	430.57	499.85	471.24 ± 133.75 ^ab^	21.01	28.05	25.03 ± 2.03 ^a^	996.9 ± 235.83 ^b^	1878.8 ± 289.72	2892.52 ± 186.31	3580.97 ± 427.6 ^ab^	861.36
H	Guards	Snarling	-	-	-	24.67	28.84	25.76 ± 1.53 ^a^	950.6 ± 191.21 ^b^	2008.48 ± 296.35	2835.99 ± 296.35	4191.21 ± 570.81 ^a^	1080.20
I	Distress, Appeal	Whimpering	75.38	597.03	575.45 ± 71.5 ^a^	20.35	29.38	27.37 ± 4.27 ^a^	1060.82 ± 254.28 ^ab^	2019.5 ± 221.21	2840.33 ± 310.44	3728.59 ± 446.12 ^ab^	889.26
J	Loneliness, Distress	Whimpering	568.07	569.74	568.75 ± 120.87 ^ab^	19.63	26.95	23.19 ± 4.93 ^a^	1088.11 ± 202.5 ^ab^	2030.16 ± 386.3	3064.48 ± 136.22	3907.17 ± 404.57 ^ab^	939.69
K	Distress	Howling	564.38	599.16	576.2 ± 113.57 ^ab^	24.71	28.13	25.92 ± 2.31 ^a^	623.22 ± 234.73 ^c^	1701.58 ± 221.25	2766.63 ± 344.12	3970.54 ± 612.2 ^ab^	1115.77
SEM					49.8			2.86	102.86	206.37	354.74	318.69	197.47
*P*-value					0.0436			0.0398	0.0489	0.1263	0.0867	0.0368	0.1283

Abbreviation: Scenarios with the same letter superscript are not significantly different (*p* > 0.05). Scenario H (snarling) is not shown as no fundamental frequency could be reliably measured.

## Data Availability

The datasets used and analysed during the current study are available from the corresponding author on reasonable request; the data are not publicly available due to privacy restrictions.
